# Precise Modeling of the Particle Size Distribution in Emulsion Polymerization: Numerical and Experimental Studies for Model Validation under Ab Initio Conditions

**DOI:** 10.3390/polym15224467

**Published:** 2023-11-20

**Authors:** Porfirio López-Domínguez, Enrique Saldívar-Guerra, María Esther Trevino, Iván Zapata-González

**Affiliations:** Centro de Investigación en Química Aplicada (CIQA), Saltillo 25294, Mexico; porfirio.lopez.ps@ciqa.edu.mx (P.L.-D.); esther.trevino@ciqa.edu.mx (M.E.T.); ivan.zapata@ciqa.edu.mx (I.Z.-G.)

**Keywords:** emulsion polymerization, population balance equations, sustainable process, particle size distribution

## Abstract

The particle size distribution (PSD) in emulsion polymerization (EP) has been modeled in the past using either the pseudo bulk (PB) or the 0-1/0-1-2 approaches. There is some controversy on the proper type of model to be used to simulate the experimental PSDs, which are apparently broader than the theoretical ones. Additionally, the numerical technique employed to solve the model equations, involving hyperbolic partial differential equations (PDEs) with moving and possibly steep fronts, has to be precise and robust, which is not a trivial matter. A deterministic kinetic model for the PSD evolution of ab initio EP of vinyl monomers was developed to investigate these issues. The model considers three phases, micellar nucleation, and particles that can contain n≥0 radicals. Finite volume (FV) and weighted-residual methods are used to solve the system of PDEs and compared; their limitations are also identified. The model was validated by comparing predictions with data of monomer conversion and PSD for the batch emulsion homopolymerization of styrene (Sty) and methyl methacrylate (MMA) using sodium dodecyl sulfate (SDS)/potassium persulfate (KPS) at 60 °C, as well as the copolymerization of Sty-MMA (50/50; mol/mol) at 50 and 60 °C. It is concluded that the PB model has a structural problem when attempting to adequately represent PSDs with steep fronts, so its use is discouraged. On the other hand, there is no generalized evidence of the need to add a stochastic term to enhance the PSD prediction of EP deterministic models.

## 1. Introduction

Ab initio emulsion polymerizations (EPs) are complex multiphase processes composed of key ingredients: water, monomers, initiator, surfactant, chain transfer agent, inhibitor, and buffer [1,2]. A commercial recipe may contain more than twenty ingredients [1]. Some relevant products of industrial EP include adhesives, styrene-butadiene rubber (SBR) for carpet backing, textile fibers, high impact strength materials, emulsion paints, bone marrow transplantations, drug delivery systems, conducting polymers, sealants, and cosmetic products [3]. Their average polymer characteristics can be controlled using on-line monitoring techniques such as Raman, near-infrared (NIR), and photon density wave (PDW) spectroscopy [4]. 

Population balance equations (PBEs) have been extensively employed to capture distinct phenomena affecting the evolution of the particle size distribution (PSD) of entities such as cells, droplets, crystals, and polymer particles [5,6,7,8]. PBEs are mathematically a set of hyperbolic partial integro-differential equations (PDEs) whose solutions can be attained by either deterministic or stochastic numerical methods [9,10]. In EPs, the number of radicals per polymer particle may vary from zero to a maximum number ($n_{max}$), eventually leading to an intractable set of PDEs. However, some simplifications are considered to keep the problem tractable. On the one hand, some approaches neglect particles with two or more radicals: the 0-1 model (polymer particles contain either zero or one radical) and the 0-1-2 model (a maximum of two radicals per particle). On the other hand, in another approach, it is assumed that all particles of the same mass (or size) contain the same average number of radicals, $\bar{n}\left(m,t\right)$. This hypothesis is used in the pseudo-bulk (PB) approach and in an implementation using the Fokker–Planck equation (FPE) [11]. A third approach has been built considering that small particles may follow the 0-1 model, whereas large particles may obey the PB approach [12].

A brief summary of the main features of modeling approaches for emulsion homo- and copolymerizations is presented in Table 1.

The numerical solution of hyperbolic PDE’s is challenging due to the possible presence of steep fronts as in the case of the inviscid Burger’s equation representing shock-waves [22]. One way to reduce the set of PDEs to a set of ordinary differential equations (ODEs) in time is using the weighted residual method (MWR) on finite elements, in which the solution in each element is approximated with either first-, second-, or third-order polynomials of the internal coordinate (mass or diameter particle) with time-dependent coefficients [23]. Both the orthogonal collocation on finite elements method (OCFE) and the Galerkin methods, two kinds of MWR, are widely used in the estimation of PSD in EPs [5,24]. Increasing the number of elements will improve estimates of the PSD, especially if sharp fronts are expected [25]. Recently, the evolution of seeded EPs was studied by a multiscale model built with kinetic Monte Carlo, the Fokker–Planck equation (FPE), and solved with the Galerkin method, in conjunction with traditional mass and energy balances [13]. The shape of the PSD was adjusted by varying the size-dependent diffusion coefficient in the FPE. Another practical type of MWR is the method of moments (MMs), which is particularly useful for the computation of relevant average properties such as the number average particle diameter (${\bar{D}}_{p}$) and the average number of radicals per particle ($\bar{n}$). Recently, a model based on the MMs for the 0-1-2 model has captured the effect of the number of reactors on the monomer conversion, molar mass, and copolymer composition of emulsion copolymerization of styrene-butadiene rubber and acrylonitrile-butadiene rubber in a train of CSTRs [17].

Another technique that has been successfully used to solve the PDE’s representing the PSD in EP is the finite volume (FV) method with a semi-discrete high-resolution scheme that has been implemented to solve the 0-1 and 0-1-2 approaches [19,20]. Such models accounted for homogenous nucleation and coagulation rates and were utilized, for instance, to analyze the effect of initiator and surfactant concentrations on the broadness of the PSD of poly(vinyl acetate) [20]. In another work, it was shown that the 0-1-2 and PB models produce similar results for seeded polymerizations, whereas the 0-1 model underestimates the growth rate [19]. As pointed out by Vale and McKenna, finite differences (FE) and FV methods are closely related, although FV may present some advantages [26].

In the last 2–3 decades, some groups have studied and contrasted numerical and structural aspects of PSD modeling in EP, as well as their interactions. The hyperbolic nature of the PDE’s may lead to numerical oscillations and numerical dispersion (spurious dispersion added to the solution) when steep fronts arise, but their presence and severity will depend on the specific problem being solved: ab initio or seeded polymerizations and the presence or not of coagulation. Saldívar and Ray used OCFE in a moving polymer-mass frame to solve the very challenging problem of (unseeded) ab initio batch EP without coagulation terms (which smooth out the front) using a PB model; they had to add a (small) artificial numerical diffusion term to avoid numerical oscillations that otherwise would occur at the front [14]. Unfortunately, as far as we know, no other group has attempted to solve this problem. Some other research groups have published simulation results of PSD models that did not use adequate numerical methods and therefore the simulated PSD shape generated is not reliable [11,27,28,29]; therefore, adequate numerical methods are needed to trust the solution and detect real structural deficiencies of the models used [11]. Vale and McKenna (2005) concluded that FV/FD methods coupled with high-resolution discretization schemes are the most efficient ones in terms of computation time and provide enough precision of the solution, avoiding numerical oscillations and numerical dispersion [26]. On the other hand, Hosseini et al. criticized deterministic PBE models arguing that they do not correctly predict the broadness of the distribution (theoretical predictions are too narrow compared with actual experimental data); however, to prove their point, they use a pseudo-bulk model and even though they mention that calculations were performed also with a 0-1 model, they do not show the results and apparently they only studied one case with this alternative model [11]. On these bases, these authors conclude that deterministic models are structurally inadequate and fix the problem by adding a stochastic term to the PDE, turning it into the Fokker–Planck equation. Although these groups, particularly Vale and McKenna, have studied a variety of cases (pure growth, nucleation and growth, etc.) none of them have studied in detail the numerically demanding case of a batch ab initio (nucleation) EP with no seed nor coagulation that presents a sharp front prone to numerical instabilities, at least when a PB model is used [18,19,26].

From these studies, there seems to be agreement on the fact that FE/FV methods with a high-resolution discretization scheme, as well as OCFE, are capable of providing sufficiently precise numerical solutions of the PBE models, but an ultimate test to further explore some limitations of these methods would be the solution of the ab initio batch EP with no coagulation terms (presence of steep fronts), labeled from here on as ABIBEPNC [14]. It is not clear if this steep front is present only when a PB model is used and if, as one could guess, it smoothens when a 0-1 or 0-1-2 model is used. 

With this panorama in mind, in this work, the following issues, aims, and related questions will be addressed: (i) to further test different numerical techniques, in particular FV/FD and OCFE methods, to assess their suitability and limitations to solve the PBEs arising in the modeling of the PSD in EP under different simulated conditions, in particular for the challenging ABIBEPNC problem; (ii) to investigate if the steep front in the ABIBEPNC problem, not experimentally observed, is a structural problem due to the use of a PB model and if using a 0-1 or 0-1-2 model the front is smoothed out; and (iii) to investigate if the apparently narrower broadness of the distribution predicted by theoretical models compared with experimental PSDs is a structural problem of the type of (deterministic) models used so far, and if the models need to incorporate stochastic terms, as suggested by Hosseini et al [11].

To answer these questions, in this contribution, we provide a mathematical model for the ab initio EP of vinyl monomers in batch reactors by combining PBEs and kinetic and thermodynamic models. From the modeling point of view, firstly, the model can consider a maximum number of radicals per particle, $n_{max}$, which will allow the tracking of the individual polymer particle populations, $f_{n}$, whose evolution will render the shape of the PSD. Secondly, in order to reduce numerical dispersion and dissipation, two efficient numerical methods are implemented with fixed grids. The first method is the orthogonal collocation method on finite elements (OCFE) using third-order polynomials on each element, while the second method is the FV method with a high resolution scheme. Finally, experimental PSDs measured by dynamic light scattering (DLS) for the EP homopolymerizations of Sty and MMA at 60 °C, as well as the copolymerization of Sty-MMA (50/50; mol/mol) at 50 and 60 °C, generated by our laboratory are included for comparison with model predictions. 

## 2. Materials and Methods

All reactants were obtained from Sigma-Aldrich. Sodium dodecyl sulfate (SDS, ≥98.5%) and potassium persulfate (KPS, ≥99.0%) were used as received. Styrene (Sty, ≥99%) and methyl methacrylate (MMA, 99%) were passed through a prepacked column with inhibitor remover (tert-butylcatechol or hydroquinone, respectively). Deionized water was obtained from an ionic exchange unit Milli-Q. Polymerizations were carried out in a 100-mL glass-jacketed reaction flask with a four-neck flask top, equipped with a mechanical agitation system (45° pitched four-blade impeller), a reflux condenser, and inlets for argon purging and sampling. An aqueous solution of surfactant (0.69 g of SDS in 75.0 g of water) plus 20 g of monomer were charged to the reactor. Then, the mechanical stirring (500 rpm) was started to form the emulsion and the reactor was purged with argon for 60 min to eliminate oxygen. In the last 15 min of this step, the emulsion was heated to the reaction temperature (50 or 60 ± 0.1 °C). The polymerization was started with the addition of an aqueous solution of initiator (0.06 g of KPS in 5.0 g of water). Small latex samples (~1 g) were withdrawn at given times to determine monomer conversion by gravimetric analysis. At the same given times, samples were taken to measure the particle size by dynamic light scattering (DLS) at 25 °C in a Nanotrac Wave II equipment. To prepare the samples for DLS, one drop of latex (~0.1 g) was diluted in 2 mL of water. The average particle diameter (${\bar{D}}_{p}$) used throughout this study is the Sauter average diameter ($D_{32}$) and the complete PSDs are reported as volume fraction distributions.

## 3. Mathematical Modeling

### 3.1. Kinetic Scheme

In our mathematical approach for ab initio EP, two reaction loci are considered: aqueous (aq) and polymer particle (p) phases. Monomer droplets supply monomer molecules to both the continuous and polymer particle phases. The reaction mechanism is detailed in Table 2. The species present in the aqueous phase, $I_{aq}$,$\;M_{aq}$, $R_{aq}$, $P_{aq}^{l}$, $D_{aq}^{l}$, and $T_{aq}$ represent water-soluble initiator, monomer, primary free radical, propagating radical of chain length *l*, dead polymer of chain length *l*, and chain transfer agent. $f_{n}dm=f_{n}\left(m,t\right)dm$ stands for the number of particles per liter of water in a slice *dm* of mass *m* containing *n* radicals, whereas Mic is the number of micelles per liter of aqueous phase. 

Once the initiator is added, primary free radicals and oligomeric species are generated in the aqueous phase; subsequently, they can undergo propagation, chain transfer, termination, and entry into a micelle. The latter mechanism, known as heterogeneous or micellar nucleation, leads to the creation of polymer particles with one radical, $f_{1}\left(m_{Mic},t\right)$. New or larger polymer particles are created by the entry, desorption, bimolecular termination, and rate of growth phenomena. The mass balances for all species involved in batch EP are summarized in Table 3 [15].

The first expression in Table 3 corresponds to the mass balance for $I_{aq}$, which is analytically solved. The rate of polymerization (second expression in the table), in terms of the monomer conversion, X, considers the contribution of the polymerization rate in the aqueous phase of volume $V_{aq}$ and the contribution of the polymerization rate in the polymer particles with *n* radicals of polymer mass *m* ($f_{n}\left(m,t\right)$) per liter of water, $V_{w}$. The domain of the internal coordinate is $\left[m_{min},\;m_{max}\right]$; $m_{min}$ was equated to the micelle mass and $m_{max}$ corresponds to a particle with a radius, *r*, of around 200 nm. n ranges from 0 to a maximum number of radicals, $n_{max}$. The entry of a radical ($R_{aq}$, $P_{aq}^{0}$ or $P_{aq}^{l}$) to polymer particles is calculated by the integral of the product of the polymer particle surface area, $4\pi r^{2}$, the mass transfer coefficient for the radical in the aqueous phase, $k_{mp}$, the radical concentration, and $f_{n}(m,t$). With regard to the rate of micellar nucleation, it is computed by the product of the micelle surface area, $4\pi r_{mic}^{2}$ ($r_{mic}$ is the micelle radius), the mass transport coefficient for the radical entering micelles, $k_{mm}$, and Mic. For the case of $P_{aq}^{0}$, its mass balance includes a term representing radical desorption from polymer particles, estimated by the integral of the product of the kinetic rate coefficient for the transfer to monomer reaction, $k_{tr,p}$, the monomer concentration in the polymer particles, $\left[M_{p}\right]$ and $nf_{n}(m,t$). 

The last equation of Table 3 represents the PBEs accounting for growth, entry, desorption, and termination phenomena. The left-hand side of the equation includes the accumulation and growth term, in which $\;\left(\frac{dm}{dt}\right)$, $W_{m}$, and $N_{A}$ represent the growth rate, monomer molar mass, and Avogadro number, respectively. The right-hand side for the mass balance of $f_{n}\left(m,t\right)$ sums the contribution of radical entry from the aqueous phase to the particles, desorption of radicals from the particles, and bimolecular termination of radicals in the particles with rate coefficients, e, $d_{e}$, and $\frac{V_{w}k_{t}}{2v_{p}}$, respectively, where $v_{p}\left(m,t\right)$ is the volume of the polymer particle. The calculation of the desorption coefficient includes the frequency of generation of monomeric radicals for monomer *i*, $g_{i}$, and the probability that a monomeric radical will desorb before undergoing chemical reaction, $\psi_{i}$, the diameter of the particle, $d_{p}$, a partition coefficient between the aqueous and polymer particle phases, $m_{di}$, and the diffusion coefficients of monomeric radicals in the aqueous phase and particles, $D_{wi}$ and $D_{pi}$, respectively. 

Additionally, the following list of assumptions is considered in the mathematical model:

(a) The batch reactor operates under isothermal conditions and is perfectly mixed;
(b) The minimum mass size is equal to the micelle mass $\left(m_{Mic}\right)$;
(c) Instantaneous thermodynamic equilibrium for the monomer partitioning in the phases is assumed. The partition coefficients model is implemented to calculate the monomer concentration in all phases;
(d) A Langmuir-type adsorption isotherm is employed to estimate the amount of surfactant adsorbed on the polymer particles’ surfaces;
(e) Kinetic rate coefficients are not chain-length dependent and are the same in both the aqueous and polymer particle phases;
(f) The Trommsdorff–Norrish effect is accounted for the termination rate coefficient;
(g) Coagulation effects and homogeneous nucleation are neglected;
(h) A maximum number of radicals per particle, $n_{max}=2$, is initially considered.

### 3.2. Emulsion Binary Copolymerization of Vinyl Monomers

The proposed mathematical model was extended to the case of binary copolymerization following the pseudo-homopolymerization approach [30]. To this end, the apparent kinetic rate coefficients were modified, accounting for the fraction of type i radicals ($\varphi_{i}$) and the molar fraction of monomer j, ($f_{mj}),\;i,\;j=1$ and 2. $F_{mj}$ is the instantaneous copolymer composition for monomer j. The expressions used in the pseudo-homopolymerization approach are presented in Table 4.

### 3.3. Monomer Partitioning Equations

The partition coefficients for the monomer are defined by Equations (1) and (2) [15,31].

(1) $$K_{dw}=\frac{\left[M_{d}\right]}{\left[M_{aq}\right]}=\frac{\varphi_{M,d}}{\varphi_{M,aq}},$$

(2) $$K_{pw}=\frac{\left[M_{p}\right]}{\left[M_{aq}\right]}=\frac{\varphi_{M,p}}{\varphi_{M,aq}},$$

$\varphi_{Md}$, $\varphi_{Mp}$, and $\varphi_{Mw}$ are the volume fraction of monomer in the monomer droplets, polymer nanoparticles, and aqueous phase, respectively. The global mass balances for monomer, polymer, and water are established assuming volume additivity, (see Equations (3)–(7)).

(3) $$\varphi_{Md}V_{d}+\varphi_{M,aq}V_{aq}+\varphi_{M,aq}V_{p}=V_{M},$$

(4) $$\varphi_{water,aq}+\varphi_{M,aq}=1,$$

(5) $$\varphi_{pol,p}+\varphi_{Mp}=1,$$

(6) $$\varphi_{water,aq}=\frac{V_{w}}{V_{aq}},$$

(7) $$\varphi_{pol,p}=\frac{V_{pol}}{Vp}.$$

Upon combining material and partition coefficient equations, a minimum of three equations with three unknowns ($V_{aq}$, total droplet volume, $V_{d}$, and $V_{p}$) are obtained, which can be solved by employing iterative methods such as Newton–Raphson, bisection, and hybrid methods [31,32]. The estimation of $V_{aq}$, $V_{d}$, and $V_{p}$ was efficiently achieved following the Armitage et al. algorithm [33,34]:

- Suppose initial values for $V_{aq}$, $V_{d}$, and $V_{p}$;
- Calculate the total monomer volume in the particles, $V_{M,p}$, from the following equation;
  (8) $$V_{M,p}=\frac{V_{M}}{1+\frac{K_{dw}}{K_{pw}}\;\frac{V_{d}}{V_{p}}+\frac{V_{aq}}{V_{p}K_{pw}}},$$
- Compute total monomer volume in both the aqueous phase, $V_{M,aq}$, and droplets $V_{M,d}$;
  (9) $$V_{M,aq}=\varphi_{M,p}\frac{V_{aq}}{K_{pw}},$$
  (10) $$V_{M,d}=\varphi_{M,p}\frac{K_{dw}}{K_{pw}}V_{d},$$
- New estimation for $V_{aq}$, $V_{d}$, and $V_{p}$;
  (11) $$V_{d}=V_{M,d},$$
  (12) $$V_{p}=V_{M,\;p}+V_{pol},$$
  (13) $$V_{aq}=V_{M,\;aq}+V_{w},$$
- Repeat until convergence in $V_{aq}$, $V_{d}$, and $V_{p}$ is reached.

### 3.4. Surfactant Partitioning and Micelle Concentration

The surfactant is solubilized in the aqueous phase $\left(S_{f}\right)$ and adsorbed onto the surface of polymer particles $\left(S_{a}\right)$ and monomer droplets $\left(S_{d}\right)$. The mass balance for surfactant is given by Equations (14)–(17) [17].

(14) $$S=S_{f}+S_{a}+S_{d},$$

(15) $$S_{a}=\frac{S_{p}\Gamma_{\infty }b_{s}S_{f}/V_{aq}}{1+b_{s}S_{f}/V_{aq}},$$

(16) $$S_{p}=4\pi V_{w}\sum_{n=0}^{n_{max}\;}\int_{0}^{\infty }f_{n}r^{2}\left(m\right)dm,$$

(17) $$S_{d}=\frac{3V_{d}}{a_{ed}r_{d}N_{A}},$$

Equation (15) is a Langmuir isotherm describing the adsorption of surfactant in particles with parameters $b_{s}$ and $\Gamma_{\infty }.$ In these equations, $a_{ed}$ is the area stabilized on the monomer droplets by one molecule of surfactant and $r_{d}$ is the average radius of the monomer droplets. $S_{p}$ is the total surface area of particles. A quadratic equation for $S_{f}$ is obtained by combining Equations (14)–(16) neglecting $S_{d}$ which is small (see Equation (18)):

(18) $$\left(\frac{-b_{s}}{V_{aq}}\right)S_{f}^{2}+\left(\frac{Sb_{s}}{V_{aq}}-\frac{S_{p}\Gamma_{\infty }b_{s}}{V_{aq}}-1\right)S_{f}+S=0.$$

Micelles will be present when the free surfactant concentration exceeds the critical micellar concentration, according to Equations (19) and (20):

(19) $$Mic=\frac{\left(\frac{S_{f}}{V_{aq}}-{\left[S\right]}_{cmc}\right)N_{A}a_{em}}{4\pi r_{m}^{2}}\;if\;\frac{S_{f}}{V_{aq}}\geq {\left[S\right]}_{cmc},$$

(20) $$\;Mic=0\;\;\;\;\;\;\;\;\;\;\;\;\;\;\;\;\;\;\;\;\;\;\;\;\;\;\;\;\;\;if\;\frac{S_{f}}{V_{aq}}<{\left[S\right]}_{cmc}.$$

### 3.5. Diffusion-Controlled Kinetic Rate Coefficients

As in the case of bulk FRP, the Trommsdorff–Norrish effect (autoacceleration) has been observed in EPs; the reaction locus viscosity increases as the polymer weight fraction increases. Herein, a model based on the free volume theory is incorporated. The effective kinetic rate coefficients, $k_{i}$, are corrected by a temperature-dependent factor $g_{i}$ (i=p, t,…). The analytical form of $g_{i}$ has been reported in the literature for the free radical polymerization of vinyl monomers [35,36].

### 3.6. 0-1-2 and 0-1 Model with Constant Coefficients (DM012 and DM01)

Some numerical comparisons were made using a previously reported 0-1-2 model with constant coefficients (herein referred to as DM012) [18], but without coagulation terms. The equations of the DM012 are presented in Equations (21) and (22):

(21) $$\frac{\partial }{\partial \tau }\left(\begin{array}{c} f_{0}^{*}\\ f_{1}^{*}\\ f_{2}^{*} \end{array}\right)+\frac{\partial }{\partial V^{*}}\left(\begin{array}{c} 0\\ f_{1}^{*}\\ 2f_{2}^{*} \end{array}\right)=\left(\begin{array}{ccc} -\alpha & \alpha \kappa & 2\alpha \gamma \\ \alpha & -\alpha \left(1+\kappa \right) & \alpha \left(1+2\kappa \right)\\ 0 & \alpha & -\alpha \left(1+2\kappa +2\gamma \right) \end{array}\right)\left(\begin{array}{c} f_{0}^{*}\\ f_{1}^{*}\\ f_{2}^{*} \end{array}\right)+\Omega \left(\begin{array}{c} 0\\ \delta \left(V^{*}\right)\\ 0 \end{array}\right),$$

(22) $$f^{*}=f_{0}^{*}+f_{1}^{*}+f_{2}^{*},$$

where *α*, *κ*, *γ*, and Ω are dimensionless ratios of entry to growth, desorption to entry, termination to entry, and nucleation to growth, respectively. $f_{m}^{*}$, $V^{*}$, and *τ* are dimensionless number density functions for particles having m radicals, particle volume, and time, respectively.

For the case of the 0-1 model with constant coefficients (herein referred to as DM01) [18], the following equations are used:

(23) $$\frac{\partial }{\partial \tau }\left(\begin{array}{c} f_{0}^{*}\\ f_{1}^{*} \end{array}\right)+\frac{\partial }{\partial V^{*}}\left(\begin{array}{c} f_{0}^{*}\\ f_{1}^{*} \end{array}\right)=\left(\begin{array}{cc} -\alpha & \alpha \left(1+\kappa \right)\\ \alpha & -\alpha (1+\kappa ) \end{array}\right)\left(\begin{array}{c} f_{0}^{*}\\ f_{1}^{*} \end{array}\right)+\Omega \left(\begin{array}{c} 0\\ \delta \left(V^{*}\right) \end{array}\right),$$

(24) $$f^{*}=f_{0}^{*}+f_{1}^{*}.$$

### 3.7. Numerical Implementation

**Figure 1:**
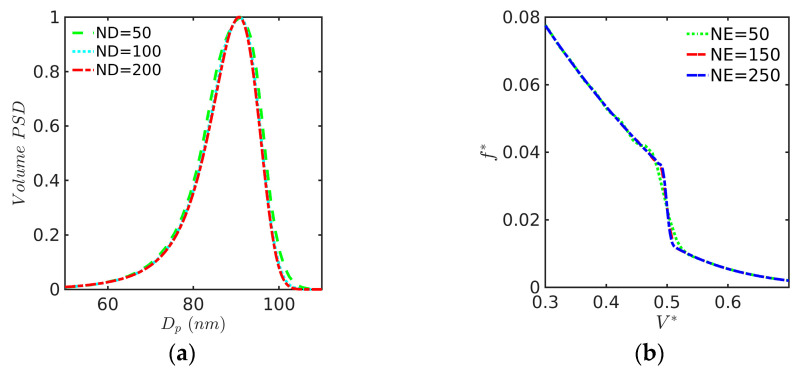
(**a**) Convergence of the FV method with a number of nodes (ND) for the PSD at t = 94 min for AIBEPNC of STY with KPS/SDS at 60 °C (Case 1 as defined below utilizing our proposed model; (**b**) convergence of the OCFE with number of elements (NE) for the AIBEPNC of hypothetical monomer using DM012 with Ω = 0.1, α=1, κ=2.11, γ=2, and τ=0.5.

The numerical methods used to calculate the PSD (the OCFE and FV methods) and polymer particle properties lead to a set of ordinary algebraic-integro-differential equations (ODEs). The set of ODEs was integrated using the ARKODE routine from the Sundials package in the C programming language with both absolute and relative tolerances set to $10^{-6}$ [37]. Most of the simulations took less than 5 min for the homopolymerization cases and about 4 hours for the copolymerization cases using a laptop or desktop computer with 16 GB of RAM. In the case of the OCFE, 150 elements were necessary to assure the convergence of the PSD, whereas 200 nodes were utilized for the FV method (see Figure 1). Definite integrals were calculated by using either Gauss–Legendre quadrature or the trapezoidal rule.

## 4. Results and Discussion

### 4.1. MWR on Finite Elements (OCFE) vs. the FV Method

**Figure 2:**
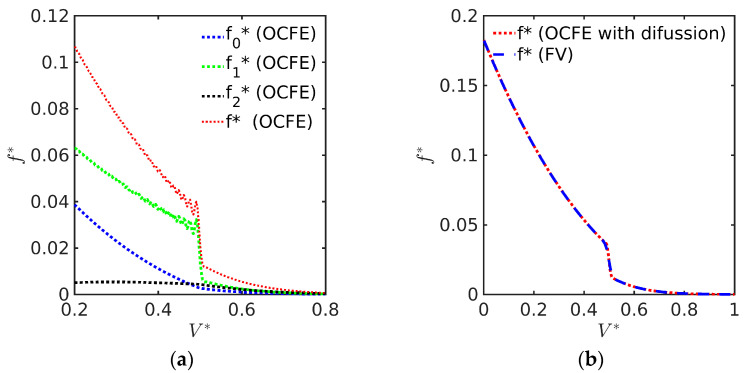
(**a**) PSD for ABIBEPNC of hypothetical monomer using DM012 with Ω = 0.1, α=1, κ=2.11, γ=2, and *τ* = 0.5, numerically solved by OCFE (NE = 200); (**b**) comparison of PSD calculated with OCFE with diffusion, $D_{a}=10^{-2}$, (NE = 200) and FV method (400 nodes) for ABIBEPNC of hypothetical monomer with Ω = 0.1, α=1, κ=2.11, γ=2, and *τ* = 0.5.

As mentioned in the Section 1, PBEs can be solved by several numerical methods including the MWR on finite elements and the FV method. However, predicted PSD calculated by some MWR may yield oscillations in the presence of nucleation sources for particulate processes [38]. For illustration purposes, a simple case involving growth and nucleation is solved by both the orthogonal collocation method on finite elements (OCFE) and the FV method using DM012 (see Section 3.6) with α=1, κ=2.11, γ=2, Ω = 0.1, and τ = 0.5. This example describes an ABIBEPNC using DM012 with a constant nucleation rate, a situation expected in the early stages of EPs, wherein a great amount of small polymer particles are present (see Figure 2a). It is observed that oscillations are generated by the OCFE method, especially for the first particles ($f_{1}$) created in the reaction volume. The oscillations arise in the $f_{1}$ distribution and are transferred to the total distribution. It has been proposed that an artificial diffusion term ($D_{a}\frac{\partial^{2}f}{\partial V^{2}}$), where $D_{a}$ is a diffusion coefficient, can be included in order to smooth out oscillations that appear in distributions with steep moving fronts [5], and this device works fine, as shown in Figure 1b that compares the OCFE + numerical diffusion solution with the FV method. It should be noted that the artificial diffusion term is not required for the FV method solved with high-resolution schemes such as the weighted essentially non-oscillatory (WENO) approximation [18].

Given these results, from here on, unless otherwise indicated, all the simulations were run using the FV technique with a high resolution scheme. Table 5 shows a summary of the numerical experiments run with different problems and methods. 

### 4.2. 0-1 Model vs. PB Approach

**Figure 3:**
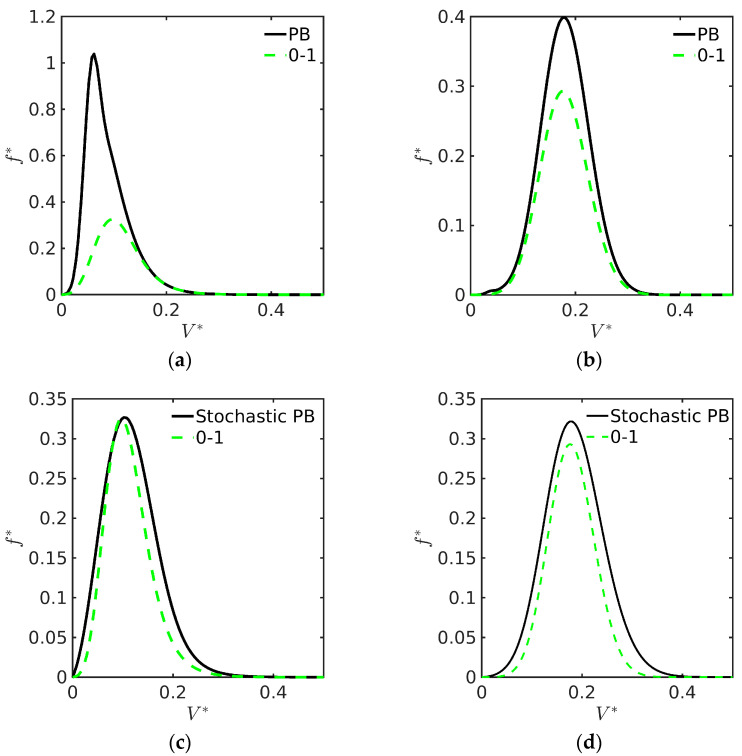
(**a**) Obtained PSDs using the DM01 and PB models with an initial exponential distribution; (**b**) obtained PSDs using the DM01 and PB models with an initial Gaussian distribution; (**c**) obtained PSDs using the DM01 and PB models with an initial exponential distribution with stochastic term; (**d**) obtained PSDs using the DM01 and PB models with an initial Gaussian distribution with stochastic term. All simulations were carried out with *α* = 1, *κ* = 2.11, and *τ* = 30 using OCFE with NE = 100.

On the one hand, the PB approach involves a single PDE to be integrated and $\bar{n}\left(V,t\right)$ is allowed to assume large values. On the other hand, PBEs considering polymer particles with n≥1 radicals per particle involve a system of n+1 PDEs whose numerical solution may be computationally demanding. As preliminary tests of the two models and before the ABIBEPNC problem is addressed, as shown below, some cases may not be well represented by the PB approach [9]. For instance, the PSD for two seeded EPs calculated by both the DM01 and the PB approaches are compared using an initial exponential distribution (see Figure 3a) and a Gaussian initial distribution (see Figure 3b). Since the PB approach requires the estimation of $\bar{n}\left(V,t\right)$, the one generated by DM01 was employed at every time step for the PB approach. The observed differences are due to the fact that particles containing $n<\bar{n}\left(V,t\right)$ radicals will grow slower than particles containing $n\geq \bar{n}\left(V,t\right)$ radicals. Moreover, it has been suggested that adding a stochastic term will improve the prediction of the PSD (see Figure 3c,d); however, this procedure requires additional information on the diffusion coefficient for each case study [11].

### 4.3. Parameter Values for Experimental Systems Studied

The kinetic coefficients and thermodynamic parameters used in the simulations are listed in Table 6 and Table 7 for Sty and MMA, respectively. The values of the kinetic rate coefficients for propagation, chain transfer to monomer, and termination reactions ($k_{p},\;k_{tr}$*,* and $k_{t}$, respectively) were equated to those reported for conventional free radical polymerization [39,40]. Regarding the constants ($K_{dw},\;K_{pw}$) used in the partition coefficients model, they were estimated from the solubility of the monomer in water and polymer [30]. The radius of a micelle (2.6 or 5 nm) has been reported in the literature [27,30]; however, our calculations were not significantly affected when this parameter was varied from 2.5 to 7 nm. The desorption-related parameters ($D_{wm},\;D_{wp}$, and $m_{d}$) have been previously estimated [30]. In the case of diffusion-controlled factors for Sty and MMA, the corresponding correlations as a function of the monomer conversion, volume fraction, or temperature were utilized [35,41]. The surfactant-related parameters ($b_{s}$, $\Gamma_{\infty }$ and $a_{em}$) and the kinetic rate coefficient for micellar nucleation ($k_{mm}=k_{mp}$), in some cases, were used as fitting parameters.

### 4.4. Simulation of Ab Initio Batch EP Homo- and Copolymerizations of Sty and MMA

The reaction conditions for EP of Sty and MMA are given in Table 8. The copolymerization experiments are directly comparable (same conditions) with those reported by Saldívar and Ray [14].

**Figure 4:**
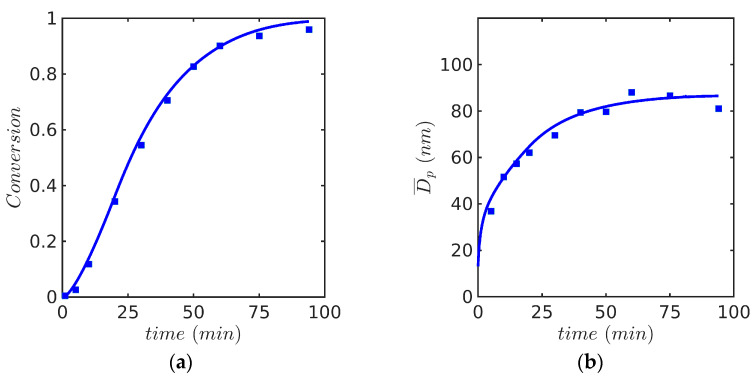

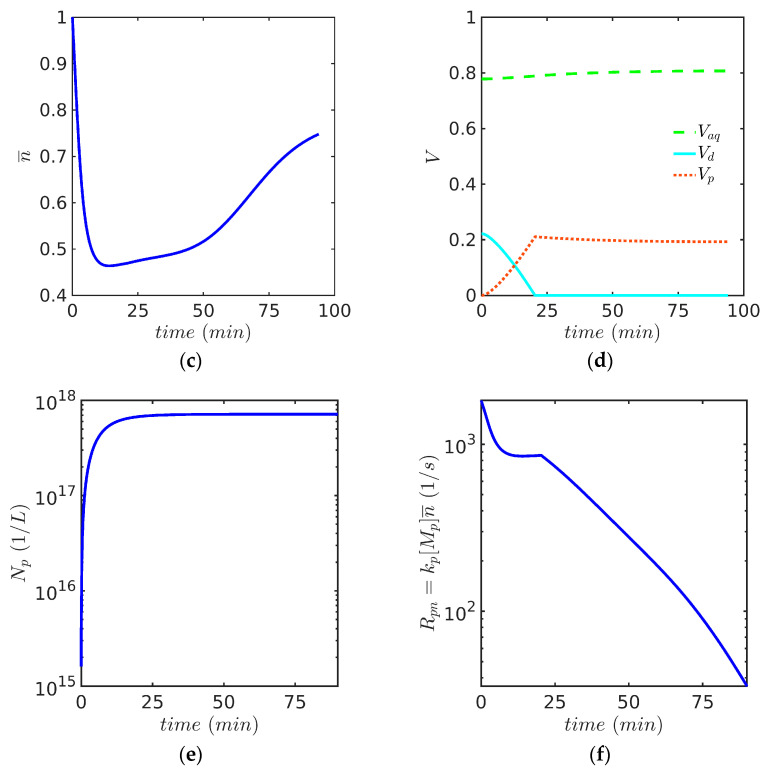
Predicted profiles of (**a**) monomer conversion, model (continuous line) vs. experiment (symbols, gravimetry), (**b**) $D_{p}$, model (continuous line) vs. experiment (symbols, DLS), (**c**) average number of radicals per particle $\bar{n}$, (**d**) volume phases against time for EP of Sty/SDS/KPS at 60° (Case 1 in Table 8) using FV method with 600 nodes and the 0-1-2 model, (**e**) total number of particles $N_{p},\;$ (**f**) rate of polymerization per particle, proportional to volume growth of the particle.

**Figure 5:**
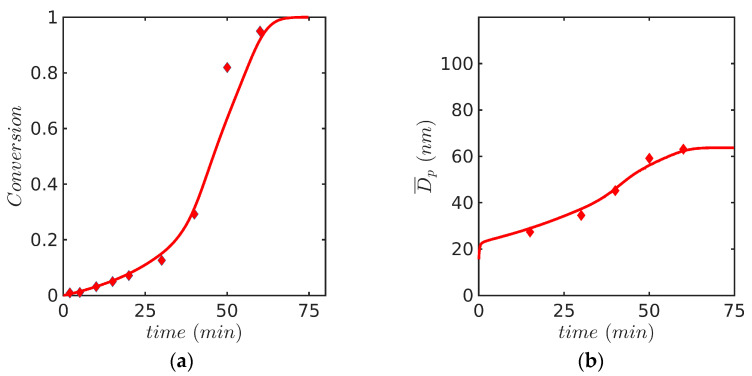

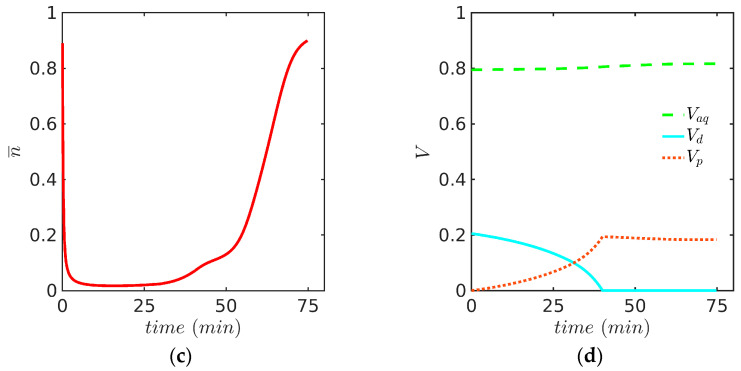
Predicted profiles of (**a**) monomer conversion, model (continuous line) vs. experiment (symbols, gravimetry), (**b**) $D_{p}$, model (continuous line) vs. experiment (symbols, DLS), (**c**) average number of radicals per particle $\bar{n}$, and (**d**) volume phases against time for EP of MMA/SDS/KPS at 60° (Case 2 in Table 8). Simulation results were obtained with the FV technique and the 0-1-2 model.

Predicted profiles of (a) monomer conversion, (b) Sauter average particle diameter, $D_{p}$, (c) average number of radicals per particle, $\bar{n}$, and (d) fractional volume phases against time for Sty and MMA are presented in Figure 4 and Figure 5, respectively. It is shown in Figure 4a and Figure 5a that the monomer conversion agrees reasonably well with experimental data for both monomers. Even though the rate of polymerization of MMA is slower than that for Sty during the first 30 min, a strong autoacceleration effect is observed for MMA and the reaction is completed at 75 min, about 25 min earlier than that for Sty. $D_{p}$ was calculated following Equation (25) [42]:

(25) $$D_{p}=D_{32}=\frac{\sum_{n=0}^{n_{max}}\int_{m_{min}\;\;}^{m_{max}}d^{3}\left(m\right)f_{n}dm}{\sum_{n=0}^{n_{max}}\int_{m_{min}}^{m_{max}}d^{2}\left(m\right)f_{n}dm}.$$

$D_{p}$ values increased with time until a constant value was reached. $D_{p}$ for Sty were about 20 units greater than those for MMA, as observed in Figure 4b and Figure 5b. With respect to the behavior of $\bar{n}$, it starts at unity since most particles initially contain a single radical due to micellar nucleation and, as the reaction proceeds, decreases until a relatively constant value is reached. $\bar{n}$ is also affected by diffusion-controlled effects (see Figure 4c and Figure 5c) and the final value of $\bar{n}$ for MMA is slightly higher than that for Sty. The evolution of the volume phases is illustrated in Figure 4d and Figure 5d; the monomer droplets phase decreases as the polymer particle phase increases, whereas the aqueous phase volume is kept constant. The aforementioned results were not changed by considering $n_{max}$ values greater than two.

Figure 4e shows the evolution of the number of particles, the slope of which indicates the nucleation rate, while Figure 4f shows the evolution of the polymerization rate per particle ($R_{pn}$), a quantity to which the rate of particle growth is proportional. During interval 1, the nucleation period, particles are being nucleated and there is competition for radicals generated in the aqueous phase between the micelles and the already formed particles. Once the nucleation period is completed (around 15–20 min), there is no more competition and all the newly formed radicals enter particles; therefore, the number of particles reaches a plateau and the particle growth rate, which originally decreased due to the competition between micelles and particles for radicals, is stabilized until the end of this period and even during interval 2. Notice that interval 2 is rather short and finishes a little before 25 min when the monomer droplets disappear (see $V_{d}$ going to zero on Figure 4d). The behavior of $R_{pn}$ and the particle growth rate are closely linked to the average number of radicals in particles ($\bar{n}$) which decreases during interval 1 and is stabilized once the nucleation stops (Figure 4c). $R_{pn}$ and the particle growth rate are also strongly linked to the monomer concentration in particles, which stays nearly constant during intervals 1 and 2. When interval 2 finishes, the monomer concentration starts decreasing in the particles until it is consumed (interval 3). This explains why the polymerization rate steadily decreases from around 25 min until the end of the reaction (Figure 4f).

**Figure 6:**
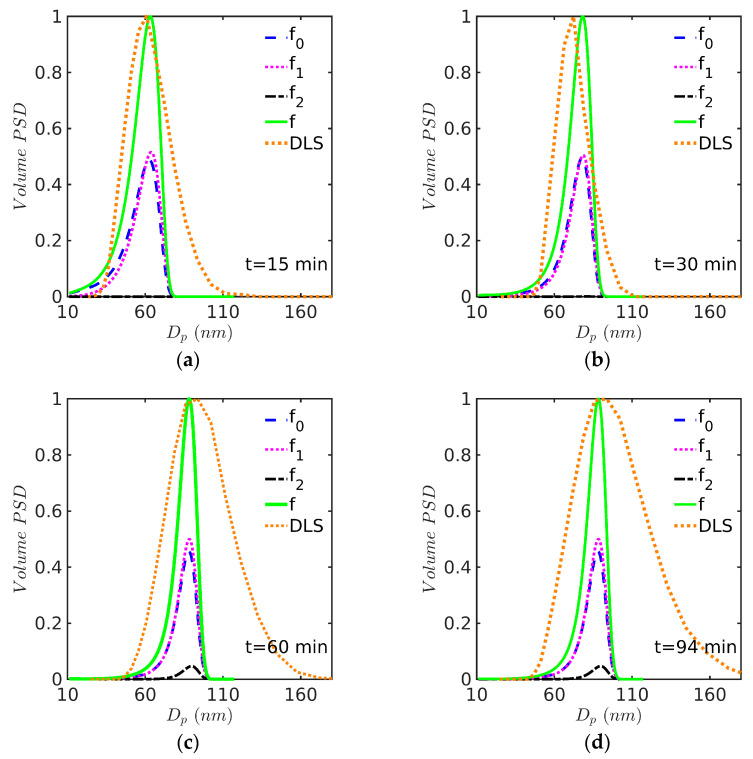
PSD at (**a**) 15, (**b**) 30, (**c**) 60, and (**d**) 90 min for EP of Sty/SDS/KPS at 60 °C (Case 1 in Table 8). Simulations run with the 0-1-2 model and solved by the FV technique.

**Figure 7:**
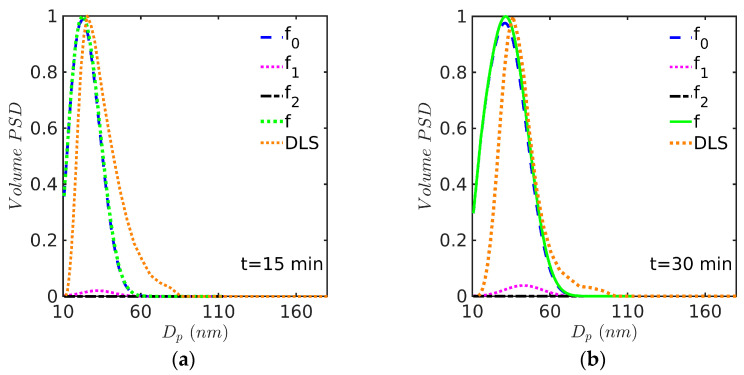

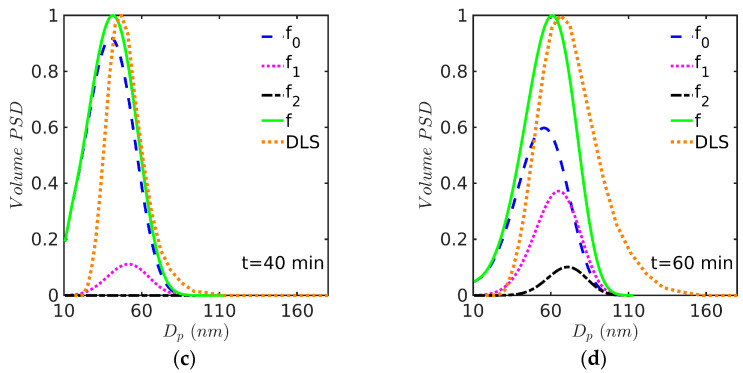
PSD at (**a**) 15, (**b**) 30, (**c**) 40, and (**d**) 60 min for EP of MMA/SDS/KPS at 60 °C (Case 2 in Table 8). Simulations run with the 0-1-2 model and solved by the FV technique.

The evolution of the calculated PSD at different times is presented in Figure 6 and Figure 7 for Sty and MMA, respectively. DLS measurements, in terms of volume, normalized at peak height were incorporated for comparison purposes. At the beginning of the reaction, small particles of about 10 nm in diameter are created through micellar nucleation; therefore, most polymer particles are concentrated at the low particle diameter region, as observed in Figure 6a and Figure 7a. In the time period of about 0 to 40 min, polymer particles with zero and one radical ($f_{0}$ and $f_{1}$) completely composed the total number of particles; after that, an important presence of polymer particles with two radicals ($f_{2}$) was observed (see Figure 6d and Figure 7d). However, DLS measurements indicate that larger polymer particles may be present in the reaction medium, a situation that is not predicted by the model. The failure of DLS to account for small particles has been pointed out by several authors in the past [43]. 

From Figure 6, it is clear that the broadness of the PSD predicted by the model in the Sty case is in general lower than the distribution estimated by DLS, as also observed by other authors for several systems. This is particularly marked for the last two distributions (at 60 and 94 min) corresponding to the largest particles, and less pronounced for the PSD at 30 min. It is important to notice, however, that the complete PSD provided by DLS is estimated using a model (the method of cumulants [44]) and it is only indicative of the true PSD, as it is well known that this estimation requires a mathematical inversion technique in which the problem is ill conditioned [45]. Also, during styrene EP, it is common to find the formation of coagulum around the stirrer, which suggests that limited coagulation is present that could lead to broader experimental PSDs; this could be accounted for by including coagulation terms in the model. 

In general, the PSDs estimated by DLS must be taken with caution. Although most of the publications cited in the introduction resort to DLS to estimate the complete PSD, it is debatable how suitable this technique is to provide a reliable estimation of this latex feature; however, most of the existing evidence points to the conclusion that, for monomodal latexes, DLS can provide a reasonably good estimation of the PSD [11,26,46]. Since our work discusses issues raised by authors that used DLS to measure the PSD, it was decided to also use this technique at this stage of our research, in spite of its limitations. Nevertheless, recognizing that there is still debate as to how reliable DLS is for estimating the latex PSD and that this subject by itself deserves a detailed separate analysis, comparisons with TEM measurements will be carried out in a future publication.

In the case of MMA (Figure 7), the experimental and model-predicted curves have similar (apparent) broadness (at least for the first three curves), although the DLS curves are shifted towards higher values, which is not surprising given the tendency of DLS to underestimate the contribution of small particles, as mentioned above. 

### 4.5. Simulation of Batch Emulsion Copolymerization of MMA with Sty

**Figure 8:**
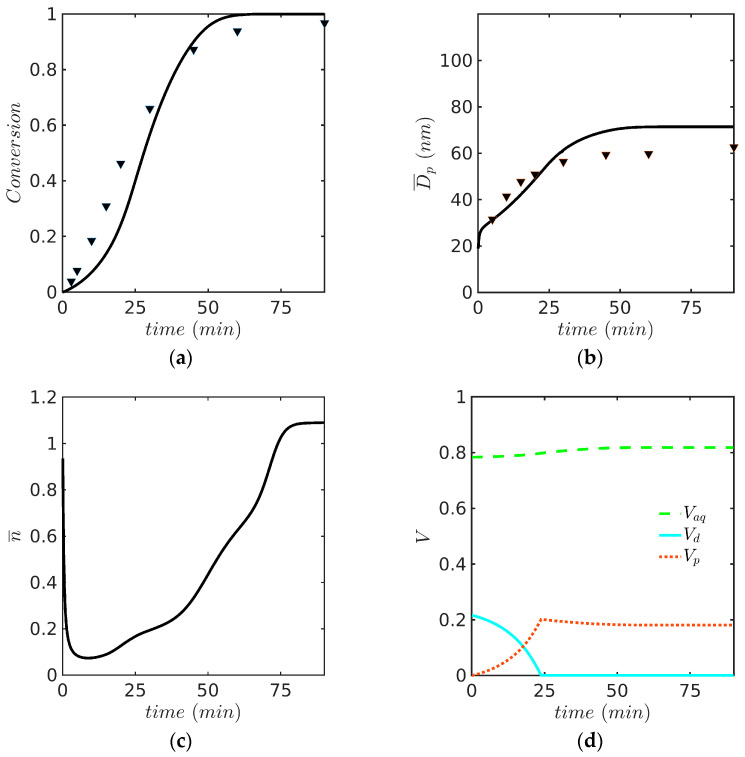
Predicted profiles of (**a**) monomer conversion, model (continuous line) vs. experiment (symbols, gravimetry), (**b**) $D_{p}$, model (continuous line) vs. experiment (symbols, DLS), (**c**) $\bar{n}$, and (**d**) volume phases against time for EP of Sty-MMA/SDS/KPS at 60° (Case 3 in Table 8). Simulated predictions with the 0-1-2 model.

This system is particularly interesting because it reproduces the experiments performed by Saldívar and Ray and their modeling results using a PB model solved with OCFE in a moving mesh domain with numerical diffusion [14]. For the emulsion copolymerization of MMA(1) with Sty(2), reactivity ratios ($r_{1}=0.46$ and $r_{2}=0.52$) were obtained from the literature [30] (notice that in this paragraph, the order of monomers used in Saldívar and Ray for the reactivity ratios is used for easy comparison). The kinetic rate coefficients used in the simulation are identical to those reported in Table 6 and Table 7. Predicted profiles of (a) monomer conversion, (b) Sauter average particle diameter, $D_{p}$, (c) average number of radicals per particle, $\bar{n}$, and (d) fractional volume phases against time are presented in Figure 8 for the experiment at 60 °C. Even though the simulations results follow the experimental trends, the monomer conversion and $D_{p}$ are somewhat overestimated at the high monomer conversion range, as observed in Figure 8a,b.

**Figure 9:**
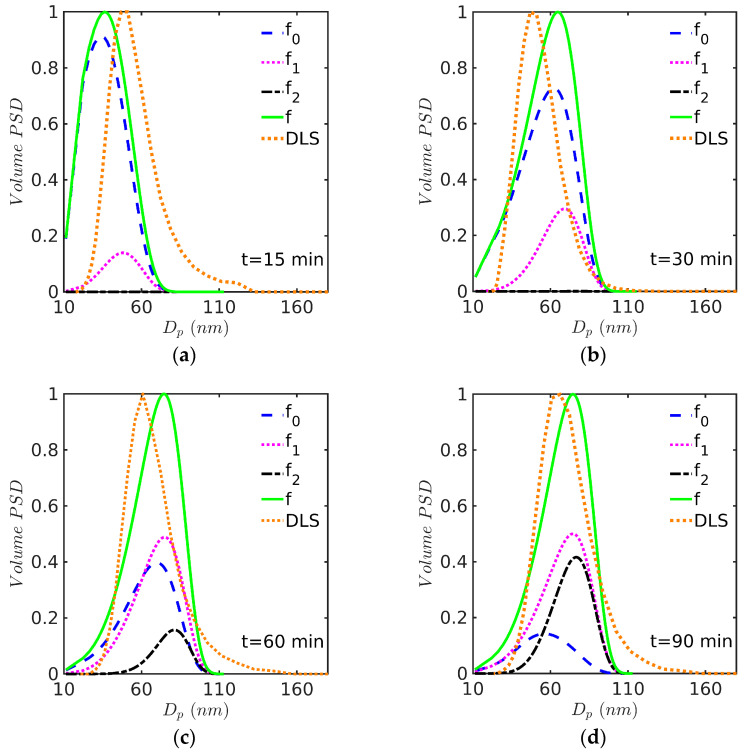
PSD at (**a**) 15, (**b**) 30, (**c**) 60, and (**d**) 90 min for EP of Sty-MMA/SDS/KPS at 60 °C (Case 3 in Table 8). Simulations run with the 0-1-2 model and solved by the FV technique.

Notably, in this case (60 °C), the broadness of the experimental and model-predicted PSDs are visually similar, although the initial experimental (DLS) curve (a) is shifted towards higher diameter values. Another interesting feature of the PSDs in Figure 9 is that the experimental DLS curves show tails at the high-diameter end of the distribution, while the theoretical curves exhibit higher densities than the experimental ones at low diameters.

**Figure 10:**
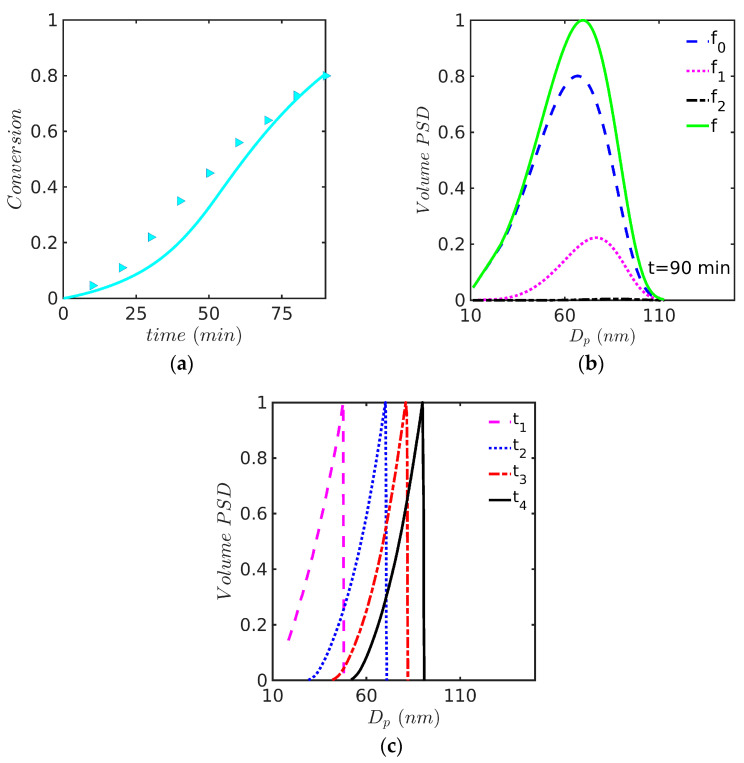
(**a**) Monomer conversion against time using the 0-1-2 model and the FV method with 200 nodes (continuous line) vs. experimental data (symbols, gravimetry), (**b**) PSD for EP of Sty-MMA /SDS/KPS at 50 °C (Case 4 in Table 8) using the 0-1-2 model and the FV method with 200 nodes, and (**c**) evolution of PSD with time using the PB model and OCFE on a moving polymer mass domain with numerical diffusion (redrawn from Figure 4 from reference [14]).

The last case study deals with the EP of Sty-MMA at 50 °C. The simulations of the monomer conversion and the PSD are presented in Figure 10. This case had been previously studied using the PB model solved by the OCFE method in a moving mesh with numerical diffusion, whose predicted PSD profile showed a sharp front (see Figure 10c redrawn from ref. [14]). In contrast, it is shown in Figure 10b that a smooth profile is obtained with the 0-1-2 model. This case addresses the second issue raised in the introduction, that of steep fronts in the ABIBEPNC problem exhibited by the PB model but not experimentally observed. As shown in the figure, this is a structural problem associated with the use of a PB model; once a 0-1-2 model is used, the front predicted by the model is smoothed out. This is apparently due to the interchange of particles between the 0-1-2 categories that move from one to another depending on the entry, desorption, or termination events occurring in particles of a given category; the interchange of categories will mean that a specific particle will grow faster or slower during different times, tending to average out the rate of growth and, therefore, smoothen out the front. 

## 5. Conclusions

In this work, we built a deterministic mathematical model to analyze the polymer reaction kinetics and PSD development of ab initio EP of vinyl monomers. The model combines kinetic and thermodynamic models with population balance equations. Four experimental cases obtained from our laboratory were addressed involving homopolymerization of Sty and MMA using sodium SDS/KPS at 60 °C, as well as copolymerization of Sty-MMA at 50 and 60 °C, in a 100 mL reactor operated under batch conditions. The computation times were relatively short for the homopolymerization cases, whereas the copolymerization cases required considerably larger CPU times (4–5 h). The model quantitatively matched quite well with the experimental data of monomer conversion and Sauter average particle diameter with minimal parameter fitting. The study allowed us to make progress in answering the issues defined in the introduction of this work. Regarding the numerical techniques to solve the PBE equations, our findings confirm those of previous researchers; the FV method with a high resolution scheme provides non-oscillatory computation-time-efficient solutions for 0-1 and 0-1-2 models; however, this technique is not capable of solving the challenging ABIBEPNC problem when a PB model is used. On the other hand, OCFE on a fixed mesh with an artificial numerical diffusion term can provide non-oscillatory solutions for 0-1 and 0-1-2 models, but it cannot solve the ABIBEPNC problem with a PB model unless a moving mass domain is used. More numerical experiments are needed to have a complete map of the applicability and limitations of the available numerical techniques for this type of problems. Concerning the steep front exhibited by the solution of the ABIBEPNC problem with a PB model, which is not experimentally observed, we conclude that this is a structural problem associated with the use of a PB model consisting of a single hyperbolic PDE; when a 0-1 or 0-1-2 model is used, the front is smoothed out due to the exchange of particles among the different classes. Regarding the last issue investigated, partially challenging the conclusion of Hosseini et al., we conclude that for some problems, the broadness of the model-predicted PSD is similar to the experimental one, although for some other cases, the predicted PSD seems to be narrower than the experimental one. However, it seems premature to conclude that this discrepancy requires a stochastic term to fix the problem, especially considering the limitations of experimental PSDs estimated by DLS. Our comparisons of model-predicted and experimental PSDs have been based on distributions estimated by DLS. These will have to be confirmed by TEM measurements that are currently being conducted in our lab and will be discussed in a future publication. Although some comparisons of experimental PSDs estimated by DLS and electron microscopy (scanning and transmission) have been made [11,29], this is limited to a few cases, so additional comparisons between these techniques should be made to reach robust conclusions.

In general, when using 0-1 and 0-1-2 models, the PSD exhibits smooth fronts that follow the experimental trends. A value of $n_{max}=2$ was initially considered; however, greater values for $n_{max}$ did not improve the observed trends. If realistic PSDs are to be predicted, the use of PB models is discouraged as they suffer from a structural problem associated with the use of a single hyperbolic PDE to represent the PSD, which is more evident in some problems, e.g., the ABIBEPNC problem.

## Figures and Tables

**Table 1:** Comprehensive mathematical models for emulsion homo- and copolymerizations of vinyl/divinyl monomers.

Monomers	Reactor Type	PSD Model	Numerical Method	Monomer Equilibrium Model	Experimental PSD	Reference
Vinyl acetate	Semibatch	FPE	Galerkin	Solubility data	-	[13]
Styrene-methyl methacrylate	CSTR	PB	OCM	Partition coefficients	-	[14,15]
Vinyl acetate-butyl acrylate	Semibatch	PB	OCM	Partition coefficients	Yes	[16]
Sty-butadiene, acrylonitrile-butadiene	CSTR	0-1-2	Method of moments	Partition coefficients	-	[17]
Vinyl chloride	CSTR	0-1-2	Finite volume	(not reported)	-	[18]
Vinyl chloride	Batch	0-1-2	Finite volume	Solubility data	-	[19]
Vinyl chloride	Batch	0-1	Finite volume	Partition coefficient	Yes	[20]
Styrene, butyl acrylate	Batch	0-1/PB	Backward finite difference	Solubility data	Yes	[12]
Styrene	Semibatch	FPE	Finite volume	Morton equation	Yes	[11]
Styrene-butyl acrylate	Batch and semibatch	PB	Fixed pivots	Partition coefficients	Yes	[21]

**Table 2:** Kinetic mechanism for EP of vinyl monomers.

Reaction	Representation
Initiator dissociation	$I_{aq}\overset{fk_{d}}{\to }\;2\;R_{aq}$
Chain initiation	$R_{aq}+M_{aq}\overset{k_{ri,aq}}{\to }P_{aq}^{1}$
Propagation	$P_{aq}^{l}+M_{aq}\overset{k_{p,aq}}{\to }P_{aq}^{l+1}$, l=1,…., cr−1
Chain transfer to monomer	$P_{aq}^{l}+M_{aq}\overset{k_{tr,aq}}{\to }D_{aq}^{l}+P_{aq}^{0}$, l=1,…., cr−1
Chain transfer to chain transfer agent (CTA)	$P_{aq}^{l}+T_{aq}\overset{k_{T,aq}}{\to }D_{aq}^{l}+P_{T,aq}^{0}$, l=1,…., cr−1
Termination by coupling	$P_{aq}^{l}+P_{aq}^{m}\;\overset{k_{tc,aq}}{\to }\;D_{aq}^{l+m}$, l=1,…., cr−1
Termination by disproportionation	$P_{aq}^{l}+P_{aq}^{m}\overset{k_{td_{ij}}^{w}}{\to }\;D_{aq}^{l}+D_{aq}^{m}$, l=1,…., cr−1
Micellar nucleation	$P_{i,aq}^{l}+Mic\overset{k_{mic}}{\to }f_{1}$
Entry into nanoparticles	$P_{i_{w}}^{l}+f_{n}\overset{k_{entry}}{\to }f_{n+1}$
Exit from nanoparticles	$f_{n}\overset{k_{des}}{\to }f_{n-1}+P_{aq}^{0}$
Bimolecular termination of radicals in polymer particles	$f_{n}\overset{k_{t}}{\to }f_{n-2}$

**Table 3:** Mass balances for species involved in batch EP.

Species	Mass Balance
Initiator	$\left[I_{aq}\right]={\left[I_{aq}\right]}_{0}\exp\left(-k_{d,aq}t\right)$
Monomer conversion	$M_{0}\frac{dX}{dt}=k_{p,aq}\left[M_{aq}\right]\left[P_{aq}\right]V_{aq}+k_{p,p}\left[M_{p}\right]\frac{V_{w}}{N_{A}}\sum_{n=1}^{n_{max}}\int_{m_{min}}^{m_{max}}nf_{n}dm\;\;$
Primary free radicals	$\begin{array}{c} \frac{1}{V_{aq}}\frac{d\left([R_{aq}]V_{aq}\right)}{dt}=2fk_{d,aq}\left[I_{aq}\right]-k_{ir,aq}\left[M_{aq}\right]\left[R_{aq}\right]-4\pi r_{mic}^{2}k_{mm}\left[R_{aq}\right]Mic-k_{t,aq}\left[R_{aq}\right]\left[R\right]-\\ V_{w}\sum_{n=0}^{n_{max}}\int_{m_{min}}^{m_{max}}4\pi r^{2}\left(m\right)f_{n}\left(m\right)k_{mR}\left[R_{aq}\right]dm;\;\left[R\right]=\left[R_{aq}\right]+\left[P_{aq}^{0}\right]+\sum_{l}\left[P_{aq}^{l}\right] \end{array}$
Monomer radicals balance	$\begin{array}{c} \frac{d\left(\left[P_{aq}^{0}\right]V_{aq}\right)}{dt}=\frac{V_{w}}{N_{A}}\overset{n_{max}}{\underset{n=1}{\sum }}\int_{m_{mic}}^{m_{max}}k_{tr,p}\left[M_{p}\right]nf_{n}\left(m,t\right)dm-k_{p}\left[M_{aq}\right]\left[P_{aq}^{0}\right]V_{aq}-4\pi r_{mic}^{2}k_{mm}\left[P_{aq}^{0}\right]MicV_{aq}\\ -k_{t,aq}\left[P_{aq}^{0}\right]\left[R\right]V_{aq}-V_{w}\overset{n_{max}}{\underset{n=0}{\sum }}\int_{m_{min}}^{m_{max}}4\pi r^{2}\left(m\right)f_{n}\left(m\right)k_{mR}\left[P_{aq}^{0}\right]dm \end{array}$
Polymeric radicals	$\begin{array}{c} \frac{d\left([P_{aq}^{l}]V_{aq}\right)}{dt}=k_{p,aq}\left[M_{aq}\right]\left(\left[\left[P_{aq}^{l-1}\right]-P_{aq}^{l}\right]\right)V_{aq}-4\pi r^{2}k_{mm}\left[P_{aq}^{l}\right]MicV_{aq}-k_{t,aq}\left[P_{aq}^{l}\right]\left[R\right]V_{aq}\\ -V_{w}\overset{n_{max}}{\underset{n=0}{\sum }}\int_{m_{min}}^{m_{max}}4\pi r^{2}\left(m\right)f_{n}\left(m\right)k_{mR}\left[P_{aq}^{l}\right]dm \end{array}$
Number of particles	$\begin{array}{c} \frac{\partial f_{n}V_{w}}{\partial t}+\frac{\partial \left(\frac{dm}{dt}\right)V_{w}f_{n}}{\partial m}=N_{A}V_{w}e\left\{f_{n-1}-f_{n}\right\}+d_{e}\left\{\left(n+1\right)f_{n+1}-nf_{n}\right\}+\frac{V_{w}k_{t}}{2v_{p}}\{\left(n+2\right)\left(n+1\right)f_{n+2}-\\ n\left(n-1\right)f_{n}\},\;\frac{dm}{dt}=\frac{k_{pp}n\left[M_{p}\right]W_{m}}{N_{A}},\;e=4\pi r^{2}k_{mp}\left[R\right],\;d_{e}=g\psi_{i},\;g=k_{tr}\left[M_{p}\right],\;v_{p}=\frac{4}{3}\pi r^{3},\;\psi_{i}=\\ \frac{K_{0i}}{K_{0i}+k_{pp}\left[M_{p}\right]},\;K_{0i}=\frac{12}{d_{p}^{2}}\frac{D_{wi}D_{pi}}{m_{di}D_{pi}+2D_{wi}} \end{array}$

**Table 4:** Apparent kinetic rate coefficients and parameters used in the pseudo-homopolymerization approach.

Kinetic Coefficient or Parameter	Expression
$k_{p}$, $k_{t}$, $k_{tr}$, $k_{mm}=k_{mp}$	$\begin{array}{c} k_{p}=\sum_{i=1}^{2}\sum_{j=1}^{2}k_{pij}\varphi_{i}f_{mj},\;k_{t}=\sum_{i=1}^{2}\sum_{j=1}^{2}k_{tij}\varphi_{i}\varphi_{j},\;k_{trij}=\sum_{i=1}^{2}\sum_{j=1}^{2}k_{trij}\varphi_{i}f_{mj},\;\\ k_{mm}=\sum_{j=1}^{2}k_{mmj}\varphi_{j} \end{array}$
$\varphi_{1},\varphi_{2}\;\;$	$\varphi_{1}=\frac{k_{p21}\varphi_{2}f_{1}}{k_{p21}\varphi_{2}f_{1}+k_{p12}\varphi_{1}f_{2}}$, $\varphi_{2}=1-\varphi_{1}$
$f_{m1},\;f_{m2}$	$\frac{df_{m1}}{dt}=\frac{f_{m1}-F_{m1}}{1-x}\frac{dx}{dt}$, $f_{m2}=1-f_{m1},$ $F_{m1}=\frac{r_{1}f_{m1}^{2}+f_{m1}f_{m2}}{r_{1}f_{m1}^{2}+2f_{m1}f_{m2}+r_{2}f_{m2}^{2}}$

**Table 5:** Summary of results for numerical experiments with different numerical techniques (√ indicates a working solution).

Model, Problem	FV + WENO on Fixed Domain	OCFE on Fixed Domain	OCFE on Fixed Domain with Numerical Diffusion	OCFE on Moving Domain with Numerical Diffusion
PB, ABIBEPNC	No convergence	No convergence	NA	Solution √
0-1 or 0-1-2, ABIBEPNC	Solution √	Solution with initial numerical oscillations in $f_{1}$ that fade away at low conversion	NA	NA
0-1 or 0-1-2, seed + nucleation	Solution √	Solution with initial numerical oscillations in $f_{1}$ that fade away at low conversion	Solution √	NA

**Table 6:** Kinetic coefficients for the EP of styrene, T in K.

Kinetic Coefficient or Parameter	Value	Reference
$k_{d}$ (s^−1^), f	$1.8\times 10^{17}\exp\left(-37,162/T\right)$, 0.5	[36]
$k_{p,aq}=k_{p,p}$ (L mol^−1^ s^−1^)	$4.27\times 10^{7}\exp\left(-3909\;/T\right)$	[39]
$k_{tr,aq}=k_{tr,p}$ (L mol^−1^ s^−1^)	$7\times 10^{-5}k_{p}$	[36]
$k_{tc,aq}=k_{tc,p}$ (L mol^−1^ s^−1^)	$1.06\times 10^{9}\exp\left(-753\;/T\right)$	[39]
$K_{dw},\;K_{pw}$ (dimensionless)	3330, 2208	[30]
$D_{wm}=D_{wp}\left({\mathrm{dm}}^{2}{\;s}^{-1}\right)$, $m_{d}\;\left(\mathrm{dimensionless}\right)$	$3.55\times 10^{-13}$, 1	[30]
$r_{Mic}\left(\mathrm{dm}\right),\;CMC\left({\mathrm{mol}\;L}^{-1}\right)$	$5\times 10^{-8}$, $1.73\times 10^{-3}$	[30]
$b_{s}\left({L\;\mathrm{mol}}^{-1}\right)$, $\Gamma_{\infty }\left({\mathrm{mol}\;\mathrm{dm}}^{-2}\right),\;\;a_{em}\left({\mathrm{dm}}^{2}\right)$	$2\times 10^{2},\;6\times 10^{-9},\;6\times 10^{-18}$	Fitted
$k_{mm},\;k_{mp}\left(\mathrm{dm}\;s^{-1}\right)$	$1.5\times 10^{-6}$	[30]
$g_{t}$	$\begin{array}{c} \exp\left(s_{1}x+s_{2}x^{2}+s_{3}x^{3}\right),\;s_{1}=-7.14+0.0101T,\;s_{2}=-19.12+\\ 0.0352T,\;s_{3}=6.06-0.0157T \end{array}$	[41]

**Table 7:** Kinetic coefficients for the EP of MMA, T in K.

Kinetic Coefficient or Parameter	Value	Reference
$k_{p,aq}=k_{p,p}$ (L mol^−1^ s^−1^)	$2.39\times 10^{6}\exp\left(-2669/T\right)$	[39]
$k_{tr,aq}=k_{tr,p}$ (L mol^−1^ s^−1^)	$2\times 10^{-5}k_{p}$	[36]
$k_{t,aq}=k_{t,p}$ (L mol^−1^ s^−1^)	$5.2\times 10^{8}\exp\left(-697/T\right)$	[39]
$K_{dw},\;K_{pw}$ (dimensionless)	59, 42	[30]
$D_{wm},\;D_{wp}\left({\mathrm{dm}}^{2}{\;s}^{-1}\right)$	$5.5\times 10^{-10}$	[30]
$m_{d}$ (dimensionless)	1	[30]
$b_{s}\left({L\;\mathrm{mol}}^{-1}\right)$, $\Gamma_{\infty }\left({\mathrm{mol}\;\mathrm{dm}}^{-2}\right)$, $a_{em}\left({\mathrm{dm}}^{2}\right)$	$2\times 10^{2},\;6\times 10^{-9}$, 1.2$\times 10^{-17}\;$	Fitted
$k_{mm},\;k_{mp}\left({\mathrm{dm}\;s}^{-1}\right)$	$1\times 10^{-6}$	Fitted
$g_{t}$ ($V_{f}>V_{f,cr2}$,)	$0.10575\exp\left(17.15V_{f}-0.01715\left(T-273.2\right)\right),\;$ $V_{f,cr2}=0.1856-2.965\times 10^{-4}\left(T-273.2\right)$	[35]
$g_{t}$ ($V_{f}\leq V_{f,cr2}$,)	$2.3\times 10^{-6}\exp\left(75V_{f}\right)$	[35]

**Table 8:** Initial conditions for the batch EP of vinyl monomers.

Case Study	Monomer	Temperature (°C)	$M_{0}/I_{0}$	$\mathrm{Initial}\;f_{m1}$	Solid Content (%)
1	Sty	60	10.7	-	20
2	MMA	60	10.7	-	20
3	Sty(1)/MMA(2)	60	10.7	0.5	20
4	Sty(1)/MMA(2)	50	10.7	0.5	20

## Data Availability

Data are contained within the article.
